# A RapidArc planning strategy for prostate with simultaneous integrated boost

**DOI:** 10.1120/jacmp.v12i1.3320

**Published:** 2010-09-28

**Authors:** David Jolly, Dineli Alahakone, Juergen Meyer

**Affiliations:** ^1^ Wellington Blood & Cancer Centre Capital and Coast District Health Board Wellington New Zealand; ^2^ Department of Physics & Astronomy University of Canterbury Christchurch New Zealand

**Keywords:** RapidArc, prostate, planning strategy

## Abstract

Since the clinical implementation of novel rotational forms of intensity‐modulated radiotherapy, a variety of planning studies have been published that reinforce the major selling points of the technique. Namely, comparable or even improved dose distributions with a reduction in both monitor units and treatment times, when compared with static gantry intensity‐modulated radiotherapy. Although the data are promising, a rigorous approach to produce these plans has yet to be established. As a result, this study outlines a robust and streamlined planning strategy with a concentration on RapidArc class solutions for prostate with a simultaneous integrated boost. This planning strategy outlines the field setup, recommended starting objectives, required user interactions to be made throughout optimization and post‐optimization adjustments. A comparative planning study, with static gantry IMRT, is then presented as justification for the planning strategy itself. A variety of parameters are evaluated relating to both the planning itself (optimization and calculation time) and the plans that result. Results of this comparative study are in line with previously published data, and the planning process is streamlined to a point where the RapidArc optimization time takes 15±1.3 minutes. Application of this planning strategy reduces the dependence of the produced plan on the experience of the planner, and has the potential to streamline the planning process within radiotherapy departments.

PACS numbers: 87.55.x, 87.55.D, 87.55.de, 87.55.dk

## I. INTRODUCTION

Interest in rotational forms of intensity‐modulated radiotherapy (IMRT) has been increasing in recent times due to advancements in delivery technique and optimization algorithms^(^ ^1^ ^)^ followed by the clinical adoption from two major vendors: RapidArc (RA) (Varian Medical Systems, Palo Alto, CA, USA) and VMAT (Elekta, Crawley, UK) (collectively referred to as rotational IMRT). These treatments are delivered over one or several continuous arcs whilst the multileaf collimator (MLC) positions, dose rate and gantry angular velocity may be dynamically varied. A variety of planning related studies^(^ ^2^ ^–^ ^19^ ^)^ comparing three‐dimensional conformal radiotherapy (3DCRT), static gantry IMRT, rotational IMRT and sometimes TomoTherapy or proton therapy,^(^ ^4^ ^,^ ^9^ ^,^ ^14^ ^,^ ^18^ ^)^ for various clinical sites (including prostate^(^ ^3^ ^,^ ^4^ ^,^ ^18^ ^,^ ^19^ ^)^ have been previously published. In general, these studies have shown rotational IMRT to be at least comparable with static gantry IMRT (both IMRT techniques make improvements over standard 3DCRT), while many show an improved level of conformity, sparing of critical structures, and a slightly reduced level of homogeneity in the target volume. All studies so far published uphold the major selling point of rotational techniques by reducing both treatment time (TT) and monitor units (MUs) significantly, when compared with other intensity‐modulated techniques.

Rotational IMRT planning is performed through inverse planning techniques in a similar vein to that of static gantry IMRT. This is further complicated due to the increased number of dynamic variables involved during delivery. Varian's solution (based on work by Otto^(^ ^1^ ^)^ is the introduction of a new resolution‐based optimization algorithm to aid in the inverse planning process. Although the clinical advantages of rotational techniques seem to be establishing themselves, a systematic process providing a turnkey solution for the inverse planning process is yet to be established. As a result, there is a strong correlation between the experience of the planner and the resulting plan quality. The aim of this work is to provide a robust strategy or class solution,^(^ ^20^ ^)^ which streamlines the planning process and produces clinically acceptable plans, in the majority of cases. An IMRT class solution was defined by Mott et al.^(^ ^20^ ^)^ as “… a set of IMRT planning parameters (beam arrangements, dose limits and penalties) that can be applied to every patient”. In the same fashion, this strategy will concentrate on RA plans produced using the complementary planning software (Eclipse V8.5) and the progressive resolution optimizer (PRO) for intermediate and high‐risk prostate patients, with a simultaneous integrated boost (SIB). The strategy will outline a systematic and streamlined workflow diagram and will cover: the field setup, recommended starting objectives and required user interactions to be made throughout optimization. This will be followed by a comparative planning study with the more standard static gantry IMRT, but will not be used as a justification for rotational techniques in general ‐ as previously published ‐ but instead, for the methods used to produce the RA plans themselves.

## II. MATERIALS AND METHODS

**Figure 1:**
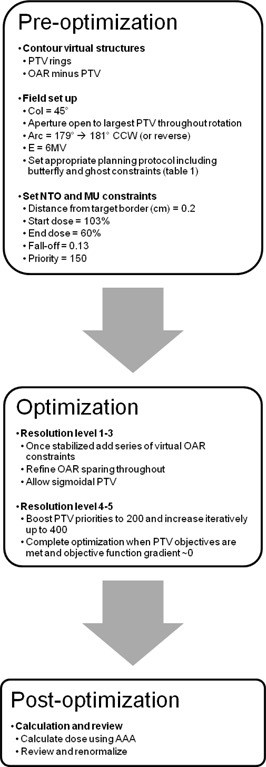
Summary of planning strategy, represented as a workflow diagram.

The following strategy is intended for the use of planning SIB RA plans for patients with intermediate and high‐risk prostate carcinoma with at least one phase including the seminal vesicles. This empirical strategy has been devised through extensive planning experience and modifications made to the many planning parameters involved across a range of patients, both adopting time‐proven static gantry IMRT inverse planning techniques and devising new RA specific techniques. In the following, the application of this strategy, which is divided into pre‐optimization, optimization and post‐optimization (see Fig. 1) is described in general terms before it is applied to a specific prostate planning protocol, namely the Conventional or Hypofractionated High Dose Intensity‐Modulated Radiotherapy for Prostate Cancer (CHHiP) trial protocol.^(^ ^21^ ^)^

### A. Planning strategy

#### A.1 Pre‐optimization

##### A.1. a) Contouring

**Figure 2:**
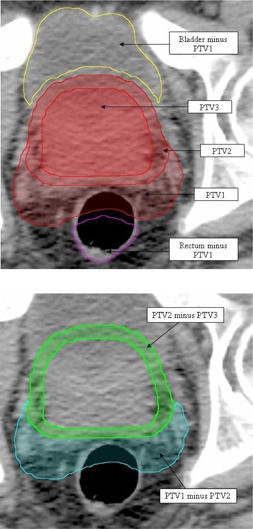
Virtual contours, as indicated by arrows: (a) showing all three PTVs and two virtual structures representing the OAR (bladder and rectum) minus the largest volume PTV, plus an extra margin of 3 mm; (b) two virtual PTV structures (PTV1 minus PTV2 (cyan) and PTV2 minus PTV3 (green)).

The first step of the planning process is contouring. In addition to standard contours, such as the body, planning target volumes (PTV) and organs at risk (OAR), additional structures are created to aid in the optimization process. This approach is similar to the use of complementary contours used for IMRT planning to improve spatial targeting of dose, henceforth referred to as virtual contours. Examples are shown in Fig. 2. In general, a virtual contour is created for any overlap between PTV and OAR (Fig. 2(a) and, if multiple dose levels exist in the target (e.g., prostate and seminal vesicles + margin →PTV1,prostate+margin→PTV2and prostate→PTV3), any overlap between the PTVs (Fig. 2(b). These virtual contours are essential for the planning strategy. The virtual OAR structures allow for improved sparing of the associated OAR while not giving conflicting optimization inputs. The PTV rings are necessary due to the fact that all PTVs occupy the same physical space and therefore nonconflicting objectives can be applied to each individually to reduce hot areas and improve homogeneity in the more superficial PTV areas.

##### A.1. b) Field setup

Once all the contours have been created, a single arc field is set with a collimator rotation of 45°.^(^ ^22^ ^)^ All RA plans require some degree of collimator rotation to reduce the cumulative effects of tongue and grove leakage throughout gantry rotation, and to allow spatial modulation in the transverse plane. The jaws are set to be open to the largest PTV throughout the entirety of the gantry rotation, with an extra margin of approximately 10 mm. The above two parameters may then be automatically optimized in Eclipse version 8.6 and above. The arc is set to run from 179° through to 181° in a counterclockwise (CCW) direction or from 181° through to 179° in a clockwise (CW) direction and the energy of the irradiating beam is 6 MV. The above field setup allows the optimization algorithm the largest range of parameters, so that the chance of the best plan being produced is maximized. Although the target volume is deep, the fact that radiation is entering the patient from all angles, a beam energy of 6 MV is adequate to produce dose coverage without the increased neutron dose that will result from higher energy beams.^(^ ^23^ ^)^

##### A.1. c) Progressive resolution optimizer

**Figure 3:**
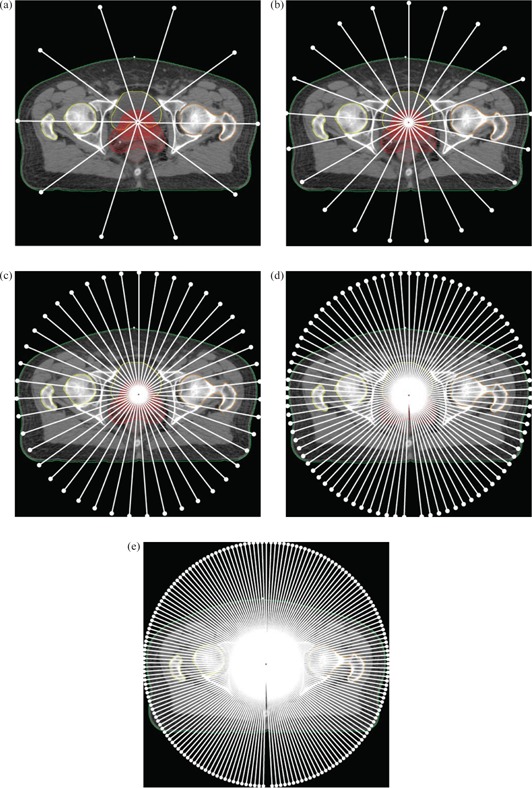
Figures (a) through (e) give a graphical representation of optimization resolution levels 1 through 5, respectively. Each level illustrates the control point distribution in relation to the patient dataset.

RA optimization and calculation in Eclipse utilizes two identical algorithms to that of standard IMRT: the dose‐volume optimizer (DVO) and anisotropic analytical algorithm (AAA), respectively. The new ‐ and unique ‐ algorithm introduced specifically for RA is the progressive resolution optimizer (PRO).^(^ ^1^ ^)^ Throughout optimization, the PRO makes changes to the dynamic delivery variables (MLC, dose rate and gantry angular velocity) iteratively, via a set of penalty functions.^(^ ^1^ ^)^ These iterations are separated into five separate resolution levels. The first represents the full arc by 10 control points (essentially static fields). This number of control points is then doubled plus one for each successive resolution level, with the final arc being represented by up to 177 control points (Fig. 3(a)–(e)). As each new control point is added, the dynamic variables are interpolated from the two neighboring points. The nature of this process means that the lower resolution levels are flexible to optimization objective change but give a coarse representation of the full arc, while the higher levels are less flexible but give a much more accurate representation of the full dynamic arc. What the introduction of this new algorithm means from a planning perspective will be outlined in the planning strategy below.

##### A.1. d) Pre‐optimization dose objectives

The inverse planning process is aided through the definition of optimization objectives. These can be either upper or lower objectives that define the input data for optimization penalty functions. Each objective corresponds to a point in the dose‐volume data space. Upper objectives give a maximum limit to the corresponding dose‐volume histogram (DVH), whilst lower objectives give a minimum. Each objective has an assigned priority related to the penalty function input, acting in a multiplicative fashion. The normal tissue objective (NTO) is a set of input parameters that defines how dose falls off outside a defined PTV, but is obviously constrained by how fast dose can physically drop off. An empirical set of these parameters, maximizing organ sparing when only the NTO is applied throughout optimization, is as follows: distance from target border=0.2 cm, start dose=103%, end dose=60%; falloff=0.13, with a priority of 150. The number of MUs required to deliver the sum of any given control point combination is directly related to the size of the corresponding MLC apertures; furthermore, small MLC apertures may be associated with dosimetric errors between AAA calculated and deliverable dose. Therefore, it is important that the maximum number of MUs is constrained throughout optimization, so as to uphold the advantages of RA in general and improve dosimetric accuracy by increasing the average beam aperture size. The strength of this objective should be high (100), but may vary depending on clinical site (an example is given in the planning comparison).

The planning strategy utilizes a set of dose objectives that is established prior to the initiation of optimization. A set of three objectives is assigned to each PTV, one lower and two upper objectives. The lower objective corresponds to 100% of the PTV volume and approximately 98% of the prescribed dose, while the first upper objective corresponds to 0% volume and 105% of the largest prescribed PTV dose. Each of these objectives initially has a low priority (P=50). The third objective is an upper objective with identical values to the above lower objective, except that it has a priority of zero. As alluded to earlier, the NTO needs a defined starting local. This position is the PTV upper objective with the lowest corresponding dose. This third objective, henceforth referred to as the ‘butterfly objective’ (due to the fact that an upper and lower objective set at the same dose‐volume point resembles a butterfly), is this starting point for the NTO. The priority of zero ensures that it has no effect on the penalty function output. In general, experience has shown that plans that employ the NTO but do not include a butterfly objective have a reduced level of PTV homogeneity and conformity and a higher maximum point dose, when compared to those that do include a butterfly objective.

An upper objective for each OAR is defined so as to push any relative hot spots away from any OAR‐PTV overlap areas. These objectives are set at the dose‐volume point corresponding to 0% of the structure volume and 98% of the prescribed dose, with a priority of P=100. An upper objective for each of the virtual PTVs created to exclude overlap with other PTVs is also assigned. These objectives should have a volume of 0% and a dose half way between the two PTV prescribed doses and have a priority of P=150.

At this point, it is useful to add a variety of ‘ghost’ objectives. These objectives will be solely used as a visual aid for the planner and will have a priority of zero, and therefore do not directly contribute to the optimization. They may directly correspond to the planning protocol OAR dose constraints (as below) or any other user‐defined DVH points of interest. Once confidence in the above strategy is established, a planning protocol can be set within Eclipse to further automate and streamline the planning approach.

#### A.2 Optimization

As previously stated, the lower optimization resolution levels are sensitive to change but give a coarse representation of the final arc, whereas the reverse is true for the higher levels. As a consequence, it is important that appropriate MLC apertures are defined early in the optimization process, as control points added further through the optimization process will be interpolated from these initial apertures. This is achieved through reserving lower levels (1–3) for sparing of critical structures while PTV coverage is concentrated on during the later stages (4–5). This is reflected through the relative priorities of the corresponding structures. Once optimization has been initialized, it is first allowed to stabilize to what is achievable through the initial starting objectives and the NTO. Following this, OAR sparing is improved upon through the addition of upper objectives added to the associated virtual OAR structures. Further objectives are not added directly to the OAR structures themselves to eliminate conflicts of objectives in areas of overlap. In saying this, improved sparing of the virtual OAR will have an associated sparing of the OAR itself, due to the spatial link shared by the two structures. These objectives are added to improve on what has already been achieved by the starting objectives alone. Highly critical structures (e.g., rectum) have assigned priorities of P=125, while other less critical structures (e.g., bladder) have a lower priority of P=50. These objectives are refined over the first three resolution levels to improve OAR sparing well within any user‐defined (ghost) constraints, using the gradient of the objective function values as a guide. It is expected that PTV coverage throughout these resolution levels will be relatively poor, with sometimes sharp sigmoidal shapes, hot volumes and even larger cold volumes, but will show improvements throughout. In saying this, it is important that the PTVs' DVHs at least cross through the upper and lower objective threshold. If this is not the case, then there may be an issue with the defined objectives (including a conflict with dose prescription) or jaw positions.

Once the optimization reaches level four, the priority of the PTV objectives are boosted to P=200 (excluding the butterfly constraint that should be frozen at zero). In general, all other objectives and their associated priorities are frozen from this point through to the completion of the optimization process. Throughout the final two resolution levels, the ability of the optimizer to meet the PTV objectives will improve. This will be at the cost of reduced OAR sparing but will be of minimal effect. If the virtual OARs' DVHs move significantly from the objectives as they were at the end of level three, then they can be relaxed slightly to give the optimizer a higher concentration on PTV coverage. Any PTV lower objective should be strictly met towards the end of level five. The priorities of the PTV objectives are increased in iterations of 50 (up to P=400), at points when the objective function gradient drops below ‐0.5. Increasing the priorities in this fashion often results in an objective function that resembles an increasing sawtooth function. The collective PTV upper objectives do not need to be strictly met but should not be far off achieving the 105% dose‐volume point. It is not of high importance that any virtual PTV structures be strictly met and may be hidden from resolution level one. The optimization is only allowed to come to completion if the total objective function gradient is close to zero and the above guidelines are met. If this is not the case, the optimization must be paused and PTV priorities increased further (if required), until the above requirements are met.

#### A.3 Post‐optimization

Following optimization, dose calculation is done using the optimized MU value (rounded to machine precision) and the AAA dose calculation algorithm with a dose grid size of 2.5 mm. The dose distribution is then evaluated and the DVHs examined for the plan's ability to meet any dose constraints. If target volume coverage does not meet ICRU 83 criteria, there may be a need to renormalize the whole plan by adjusting the plan normalization value, usually by no more than 1–2%.

### B. Planning study

The following comparative planning study serves a two‐fold purpose. Firstly, it provides a practical example of the above SIB RA planning strategy, using the CHHiP protocol. It secondly serves as an intercomparison of the produced RA plans to static gantry IMRT plans, as a justification for the methodology used to create the RA plans. The study compares RA and IMRT plans produced for 10 randomly‐selected high and intermediate risk (as defined by the CHHiP protocol; clinical stages: T1b/c, T2a/b/c or T3a, and with PSA+((Gleason score−6)*>15), previously treated patients with prostate carcinoma at the Wellington Blood and Cancer Centre. The comparison with 3DCRT has been omitted due to the results of previously published studies^(^ ^3^ ^,^ ^4^ ^,^ ^18^ ^,^ ^19^ ^)^ and the difficulty of comparing SIB and sequential boost plans.

#### B.1 Planning method

All RA and IMRT plans were produced to comply with the control arm of the CHHiP protocol, and optimized using Eclipse V8.5, on a single PC housing a quad core 2.5 GHz processor. For both types of plans, dose calculation was done with the aid of the distribution calculation framework, whereby the workload was shared by eight networked PCs identical to that used for optimization. The CHHiP protocol comes from a current prostate hypofractionation trial out of the United Kingdom.^(^ ^21^ ^)^ The control arm of this trial gives up to 74 Gy (2 Gy fractions) to three separate PTVs using a SIB. All RA plans used in the study were planned as per the above strategy. Sliding window IMRT plans consisted of five separate fields of gantry angles: 0°, 45°, 100°, 260° and 315°. These beam angles are in line with the local protocol that consistently produces plans of comparable quality to published class solutions.^(^ ^24^ ^)^ The energy of these fields was 6 MV and an identical NTO as above, was employed. A high priority was initially given to the target structures. Once PTV coverage was sufficient, virtual organ objectives (P=125) were added to spare the associated OAR as much as possible, without compromising the PTV coverage. See Table 1 for a summary of IMRT starting objectives and priorities.

**Table 1:** IMRT starting objectives. Butterfly and ghost objectives omitted.

*Structure*	*Objective*	*Volume (%)*	*Dose (Gy)*	*Priority*	*Type*
PTV1	Upper	0	77	250	PTV Upper
	Lower	100	59.2	300	PTV Lower
PTV2	Upper	0	77	200	PTV Upper
	Lower	100	71	300	PTV Lower
PTV3	Upper	0	77	200	PTV Upper
	Lower	100	74	300	PTV Lower
Rectum	Upper	0	73	100	Organ sparing
Bladder	Upper	0	73	100	Organ sparing

As per the CHHiP protocol, the gross tumor volume (GTV) is defined as the prostate only, the clinical target volume (CTV) 1 as the prostate and seminal vesicles with a 5 mm margin, CTV2 as the prostate plus a 5 mm margin, and CTV3 as the prostate only. All PTVs (1–3) add an extra 5 mm to the corresponding CTV, except that for PTV2 and PTV3 the margin in the posterior region (towards the rectum) is reduced to nil. PTVs one through three have prescribed 2 Gy equivalent doses of 54, 70 and 74 Gy, respectively (see CHHiP protocol for complete summary).^(^ ^21^ ^)^

Normal tissues structures include the rectum, bladder, femoral heads and the body (all defined as solid structures). The rectum is contoured from the anus superiorly through to the recto‐sigmoid junction. The bladder is contoured in full from base to dome (any randomly selected patient deemed to have insufficient bladder filling (< 140 cc) was rejected from the study (n=1)). All dose constraints are replicated as ghost constraints in Table 2.

**Table 2:** RapidArc starting objectives. Butterfly and ghost objectives included.

*Structure*	*Objective*	*Volume (%)*	*Dose (Gy)*	*Priority*	*Type*
	Upper	100	57.7	0	Butterfly
PTV1	Upper	0	77	50	PTV Upper
	Lower	100	57.7	50	PTV Lower
	Upper	100	69.2	0	Butterfly
PTV2	Upper	0	77	50	PTV Upper
	Lower	100	69.2	50	PTV Lower
	Upper	100	73	0	Butterfly
PTV3	Upper	0	77	50	PTV Upper
	Lower	100	73	50	PTV Lower
	Upper	0	73	100	Organ sparing
	Upper	80	30	0	Ghost
	Upper	70	40	0	Ghost
Rectum	Upper	60	50	0	Ghost
	Upper	50	60	0	Ghost
	Upper	30	65	0	Ghost
	Upper	15	70	0	Ghost
	Upper	3	74	0	Ghost
	Upper	0	73	100	Organ sparing
Bladder	Upper	50	50	0	Ghost
	Upper	25	60	0	Ghost
	Upper	5	74	0	Ghost
Femoral head	Upper	50	50	0	Ghost

#### B.2 Evaluation tools

All statistical analysis was done using a ‘two independent sample Wilcoxon rank sum test’ method. Values were deemed to be statistically different if p<0.05. Estimated RA treatment times were calculated by dividing the arc length (358°) by the average gantry rotation rate, taken from Eclipse output. Estimated IMRT treatment times were calculated through the sum of multiple factors: the time to deliver the beam (total MU/dose rate), the time to rotate through the range of field angles while traveling at 360°/min and an extra factor that accounts for the amount of time for mode up, data transfer of the MLC delivery files, error in estimated rotational time and the operator deliver time. This method was devised by Oliver et al.,^(^ ^9^ ^)^ and the time allocated for the above mentioned extra factor equals 19.1±2.2 seconds per field. The quality of PTV3 coverage was evaluated through the statistical comparison of the $V_{95}$, $D_{95}$, CI, HI, $D_{\text{max}}$ and $D_{\text{mean}}$. The $V_{95}$ is calculated as the percentage of PTV3 receiving 95% of the target dose (or 70.3 Gy). The $D_{95}$ is defined as the dose to 95% of PTV3 and the conformity index (${\text{CI}}_{95}$) defined as follows:^(^ ^25^ ^)^

(1) $$CI_{95}=\frac{V_{91}}{V_{PTV2}}$$

where $V_{91}$ is the 91% isodose volume (equivalent to 95% of the prescribed dose to PTV2) and $V_{PTV2}$ is the volume of PTV2. The conformity index gives an indication as to how conformal the minimum (95% as per ICRU) prescribed dose is to PTV2. ${\text{CI}}_{95}$ values closer to 1 indicate a higher level of conformity with any values outside the range of 0.9–1.5 deemed clinically unacceptable.^(^ ^25^ ^)^ Due to the PTV geometries and dose gradients involved, the CI of PTV2 is the only index that gives an accurate indication of the overall conformity of the plan. The homogeneity index (HI) is defined as follows:

(2) $$HI=100*\left[1-\left(\frac{D_{5\%}-D_{95\%}}{D_{mean}}\right)\right]$$

where $D_{5\%}$ is the dose to 5% of PTV3, $D_{95\%}$ is the dose to 95% of PTV3 and $D_{mean}$ is the mean PTV3 dose. HI values closer to 100% indicate more homogeneous dose coverage within the target volume. The ability of the two treatment modalities to spare critical structures will be assessed on dosimetric end points that correspond to the CHHiP protocol dose constraints (ghost constraints found in Table 2), maximum and mean dose along with OAR equivalent uniform doses (EUD). The EUD is defined as the uniform dose that will produce the equivalent radiobiological effect as the organ specific nonuniform dose distribution. This value is mathematically defined as:^(^ ^26^ ^)^

(3) $$EUD={\left(\frac{1}{N}\sum_{i}D_{i}^{a}\right)}^{\frac{1}{a}}$$

where *N* is the number of voxels within the investigated structure, $D_{i}$ represents the dose to each individual voxel within the structure, and *a* is an organ specific dose volume parameter (a=8.3 for rectum and a=2 for bladder^(^ ^19^ ^)^. Volumes of normal tissue receiving more that 2 and 5 Gy have been calculated.

## III. RESULTS

Justification for the outlined planning strategy is provided by the results of the comparative planning study, below. The 10 patient dataset average PTV3, rectum, bladder and left femoral head volumes are 94±57 (standard deviation), 48±11, 300±120 and $182\pm 31\;{\text{cm}}^{3}$, respectively. The average RA optimization and dose calculation time was 15.0±1.3 and 1.66±0.15 minutes, respectively. These respective times dropped to 8.0±0.6 and 0.6±0.1 for static gantry IMRT. The two major selling points of RA are upheld with a significant reduction in both MU and TT. On average, RA required 541±12 MU where the total for all five IMRT fields required 806±82. The average, estimated RA TT was calculated to be 65.25±0.10 seconds and, using the above methodology, the estimated IMRT TT was 243.2±11.6 seconds.

PTV3 dose evaluation parameters are summarized in Table 3. The differences between the $V_{95}$ values of RA (99.8%±0.3%) and IMRT (99.9%±0.3%) are found to be statistically insignificant. The average $D_{95}$ values were 71.6±0.3 Gy and 71.7±0.3 Gy for RA and IMRT, respectively. The average RA conformity index is statistically lower (1.08±0.06) when compared with IMRT (1.17±0.08). The HI of the IMRT plans (96.3%±0.6%) is significantly improved over the RA plans (95.8%±0.2%). The values of $D_{\text{max}}$ and $D_{\text{mean}}$ for RA and IMRT are 76.4±0.6, 75.6±0.5 Gy and 73.0±0.4, 73.2±0.3 Gy, respectively. The maximum dose is significantly more for RA, while the mean dose shows no significant difference. The combined analysis of PTV3 is in line with the majority of previously published data.^(^ ^3^ ^,^ ^4^ ^,^ ^18^ ^,^ ^19^ ^)^ RA and IMRT are comparable in most facets with the conformity of RA better than IMRT but the maximum dose within the PTV is larger. Detailed analysis of PTV1 and PTV2 is not considered here, but all 20 plans (RA and IMRT) comply with the CHHiP protocol requirements.

**Table 3:** Summary of planning study PTV3 analysis.

	*RA*		*IMRT*		
	*Average*	*Std Dev*	*Average*	*Std Dev*	*Significance*
MU	541.0	12.1	805.7	81.9	IMRT>RA
TT (s)	65.0	0.1	243.2	11.6	IMRT>RA
$V_{95}$	99.8	0.3	99.9	0.3	NS
$D_{95}$	71.6	0.3	71.7	0.4	NS
${\text{CI}}_{95}$	1.08	0.08	1.17	0.08	IMRT>RA
HI (%)	95.8	0.2	96.3	0.6	IMRT>RA
$D_{\text{max}}$	76.4	0.6	75.6	0.5	IMRT<RA
$D_{\text{mean}}$	73.0	0.4	73.2	0.3	NS

NS= not significant

**Figure 4:**
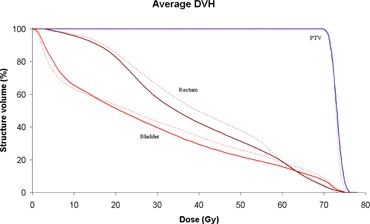
Average DVH for PTV3, rectum and bladder, comparing RA (solid lines) with static gantry IMRT (dashed lines).

As previously mentioned, the three main OARs are rectum, bladder and the femoral heads. An average DVH (of all 10 RA and IMRT patients) can be found in Fig. 4. It is seen that, on average, RA plans spare the rectum better than IMRT over the whole range of dosimetric end points. OAR dose evaluation parameters are summarized in Table 4. The high‐volume end points ($V_{80\%}-V_{30\%}$) all showed a statistical difference, while the low‐volume end points ($V_{15\%}$ and $V_{3\%}$) do not. On average, the RA plans had a significantly higher maximum dose than IMRT (73.9±0.9 and 72.7±0.8 Gy, respectively). In accordance with the fact that RA improved on dosimetric end points across the whole range, the mean dose was lower when compared with IMRT (36.2±3.8 and 39.7±3.1, respectively). The rectum EUD is also significantly lower for the RA (53.1±2.0 Gy) plans when compared with IMRT (53.9±2.0 Gy). Analysis of the bladder shows a similar trend, with RA improving on all dosimetric end points when compared with IMRT. The mean bladder dose for RA was again lower than IMRT but the maximum dose was higher. None of these differences were deemed significantly different. The bladder EUD is lower for RA (32.6±8.9 Gy) than IMRT (35.3±8.9 Gy) although, again, this difference is not statistically significant. Finally, the single dosimetric end point ($V_{50\%}$), mean and maximum dose of the (representative) left femoral head were all significantly improved for the RA plans when compared with the IMRT plans.

**Table 4:** Summary of planning study OAR analysis.

		*RA*		*IMRT*		
		*Average*	*Std Dev*	*Average*	*Std Dev*	*Significance*
Rectum	$V_{3\%}$	68.7	2.4	68.8	2.3	NS
	$V_{15\%}$	59.1	3.0	60.3	2.8	NS
	$V_{30\%}$	47.2	5.4	52.8	4.4	IMRT>RA
	$V_{50\%}$	32.2	5.2	38.1	4.3	IMRT>RA
	$V_{60\%}$	27.5	4.7	32.3	4.3	IMRT>RA
	$V_{70\%}$	23.6	4.3	29.4	6.0	IMRT>RA
	$V_{80\%}$	19.8	3.8	24.0	3.4	IMRT>RA
	$D_{\text{max}}$	73.9	0.9	72.7	0.8	IMRT<RA
	$D_{\text{mean}}$	36.2	3.8	39.7	3.1	IMRT>RA
	EUD	40.6	3.1	43.7	2.7	IMRT>RA
Bladder	$V_{5\%}$	65.9	9.1	68.2	5.2	NS
	EUD	53.1	2.0	53.9	2.0	IMRT>RA
	$V_{50\%}$	18.7	11.8	22.1	14.1	NS
	$D_{\text{max}}$	75.3	0.7	74.8	0.8	NS
	$D_{\text{mean}}$	25.7	10.0	27.5	10.8	NS
	EUD	52.8	4.6	54.0	4.6	NS
Femoral head	$V_{50\%}$	18.1	6.3	23.9	9.9	IMRT>RA
	EUD	32.6	8.9	35.3	8.9	NS
	$D_{\text{mean}}$	18.3	4.0	22.2	6.2	IMRT>RA

NS = not significant

RA spreads low doses of radiation to larger areas of normal tissue. Correlations between the induction of secondary malignancies and the volume of peripheral normal tissue receiving low doses of radiation have been reported.^(^ ^27^ ^)^ Although this is not the concentration of the paper, volume of tissue receiving low doses of radiation are reported below. The average volume of normal tissue (body minus largest PTV) receiving more than 2 and 5 Gy for RA is 8200±1300 and $5650\pm 990\;{\text{cm}}^{3}$, respectively. These values were, on average, lower for IMRT (7728±1343 and $5320\pm 891\;{\text{cm}}^{3}$), although both differences were not deemed significant.

## IV. DISCUSSION

The RA inverse planning process has both familiar and foreign aspects with regard to static gantry IMRT. The introduction of a resolution‐based optimizer means that solely employing standard IMRT class solutions and strategies does not produce the optimal plan. This is due to the fact that RA planning uses a multistep optimizing process. It is paramount to understand this process, in order to know when and how to apply appropriate planning‐based parameters, and to guide the optimizer in the desired direction. The development of new RA specific strategies is important to ensure consistency amongst plans and to streamline the planning process in general, whist producing clinically acceptable plans for a wide variety of patient anatomies. This is the first known published approach to class‐like solutions for RA planning. The authors believe that it will help departments reduce the time it takes to develop their own planning strategies when transferring from an existing static gantry IMRT program or establishing a new dynamic IMRT one. Application of the above strategy to 10 prostate patients has shown that all plans not only comply with the acceptance requirements of the planning protocol, but are also in line with previously published RA, prostate planning study data,^(^ ^3^ ^,^ ^4^ ^,^ ^18^ ^,^ ^19^ ^)^ across a broad range of both target coverage and organ sparing parameters. With the above strategy and the empirical set of parameters presented, the mean RA optimization time was 15±1.3 minutes, a justifiable increase compared to the static gantry optimization time of 8.0±0.6 minutes, when the reduction in MUs and TT is considered.

To further test the hypothesis that applying IMRT‐like class solutions to the RA planning process does not produce optimal plans and that user interaction is required (as presented above), all 10 patients were retrospectively replanned with a single planning template set and allowed to optimize without user interaction. This template contained identical objectives to those found in Table 2, except that all PTV priorities were increased to 300. A set of five upper objectives was also added to the virtual organs at risk that correlate to the average values achieved in the previous RA plans, with a priority of 125. The PTV coverage for the resulting plans was statistically comparable to that previously achieved ($D_{\text{mean}}$, $D_{\text{max}}$, ${\text{CI}}_{95}$ and HI), whilst the OAR sparing was not as good. The mean rectal dose‐volume points were lower across the range of points measured, significantly so for the $V_{50\%}$ point, as was the mean dose. This is further evidence to the point that user interactions are required to (1) assign the optimum virtual OAR objectives dependent on patient specific anatomy, and (2) define appropriate MLC apertures in the lower resolution levels. Further to this point, the average virtual OAR constraints used for the replanned optimization were based on values that had already proven successful for this small group of patients and, therefore, it can be envisioned that applying these values to a wider range of patient anatomies may produce results with even more dramatic advantages.

Although the data are not shown, further investigations revealed that the addition of a second full arc further improves OAR sparing, but the improvement was deemed too slight to justify the associated increase in both MU and TT. The above RA planning strategy has also been successfully applied in a trial on a similar yet distinct disease site where the PTV wraps around an OAR (thyroid bed and spinal cord, respectively). The strategy has also been applied to more complex treatment sites such as head & neck. Whilst the details go beyond the scope of this paper, initial findings indicate that the virtual contouring, NTO, butterfly objective and optimization user interactions can be transferred as presented here. However, more work is required to be able to determine the optimal number of arcs, collimator angle, jaw size, MU objective and avoidance sectors.

## V. CONCLUSIONS

The theoretical advantages of rotational techniques are well established, but the methods used to achieve these advantages are yet to be published. As a result, it has been shown that using the outlined field setup, starting objectives and optimization strategy, prostate RA plans can be produced in a systematic manner to produce plans that are not only clinically acceptable but in line with previously published data. The slight increase in optimization time of the PRO‐based inverse planning (compared with conventional inverse planning techniques) seems justified when other clinical advantages of RA are considered. The MU and estimated TT are significantly reduced when compared with IMRT, whilst maintaining comparable target volume coverage and an improved level of conformity. OAR sparing is also shown to be improved – and to a significant level – for both the rectum and the femoral heads. Whilst this planning strategy has only been demonstrated to work for the CHHiP protocol, it can be easily modified and implemented to suit the specific requirements of other radiotherapy departments, in an effort to treat patients with high and intermediate risk carcinoma of the prostate in a highly conformal and time‐efficient manner.

## Supporting information

Supplementary Material
