# Supplementary figures and images for: A RapidArc planning strategy for prostate with simultaneous integrated boost
Source: J Appl Clin Med Phys. 2010 Sep 28;12(1):35–49. doi: 10.1120/jacmp.v12i1.3320 (PMC5718576; doi:10.1120/jacmp.v12i1.3320)

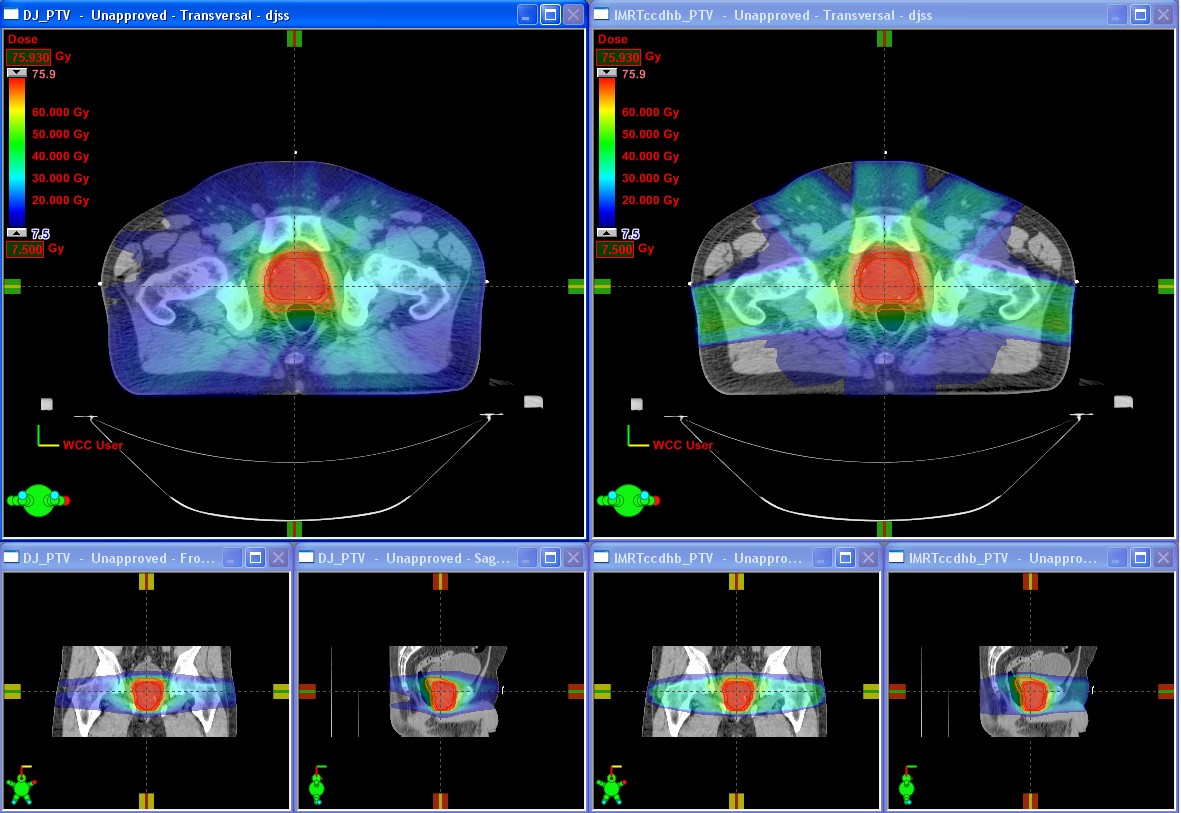

Supplement: Supplementary file 1 — Supplementary Material [file ACM2-12-035-s001.jpg]
